# Effects of Dietary Nitrates on Systemic and Cerebrovascular Hemodynamics

**DOI:** 10.1155/2013/435629

**Published:** 2013-12-25

**Authors:** Vernon Bond, Bryan H. Curry, Richard G. Adams, M. Sadegh Asadi, Richard M. Millis, Georges E. Haddad

**Affiliations:** ^1^Department of Health, Human Performance & Leisure Studies and the Cancer Center Physical Medicine & Nutrition Laboratory, Howard University, Washington, DC 20059, USA; ^2^Department of Medicine, Division of Cardiology, Howard University Hospital, Washington, DC 20060, USA; ^3^Department of Neurology, Howard University Hospital, Washington, DC 20060, USA; ^4^Department of Physiology & Biophysics, Howard University College of Medicine, Washington, DC 20059, USA

## Abstract

Cerebral blood flow dysregulation is often associated with hypertension. We hypothesized that a beetroot juice (BRJ) treatment could decrease blood pressure and cerebrovascular resistance (CVR). We subjected 12 healthy females to control and BRJ treatments. Cerebrovascular resistance index (CVRI), systolic blood pressure (SBP), total vascular resistance (TVR), and the heart rate-systolic pressure product (RPP) measured at rest and at two exercise workloads were lower after the BRJ treatment. CVRI, SBP, and RPP were lower without a lower TVR at the highest exercise level. These findings suggest improved systemic and cerebral hemodynamics that could translate into a dietary treatment for hypertension.

## 1. Introduction

Cognitive deficits are associated with dysregulation of cerebral blood flow in a wide variety of disease states [1–10]. Decreased cerebral blood flow from stroke is a prevalent complication of cardiovascular disease associated with atherosclerosis and hypertension [8]. Although it is controversial whether obesity is a disease, overweight individuals are susceptible to diabetes mellitus and most of the aforementioned cardiovascular abnormalities, defined as metabolic syndrome, also producing predilections for brain hypoperfusion [11]. In that regard, we have previously reported a predilection for decreased middle cerebral artery blood flow velocity associated with an exaggerated vasopressor response to aerobic exercise in overweight, otherwise healthy, young adult African-American university students [12]. Retired professional NFL football players with large body mass index and waist/hip ratio also seem to be at high risk for low cerebral blood flow, associated with cognitive impairment [6]. Dietary nitrate supplementation is purported to be an efficacious adjunctive treatment that decreases blood pressure [13] by the mechanism of decreasing peripheral vascular resistance [14] and may, therefore, be useful as a counter measure against brain hypoperfusion by decreasing cerebrovascular resistance. However, no studies have addressed the effects of nitrate supplementation on blood pressure-flow indexes of brain perfusion. Therefore, we designed this study to test the hypothesis that dietary nitrate supplementation with beetroot juice may increase middle cerebral artery blood flow velocity and may decrease a Doppler ultrasonographic index of cerebrovascular resistance in healthy young adult African-American female university students.

## 2. Methods

This study used a crossover design where participants were randomly assigned to receive either a placebo control orange juice or a beetroot juice treatment on separate days.

### 2.1. Subjects

Twelve healthy young adult women who were physically active but not exercise trained volunteered to participate in this study. None of the subjects were regular users of tobacco products or consumers of alcohol. They were all disease-free and had no medical conditions. The procedures outlined in the study were approved by the Institutional Human Participants Review Board of Howard University. After the experimental procedures and study risks were explained, all subjects gave their written informed consent before commencement of the study. Percentage of body fat was measured by dual energy X-ray absorptiometry (GE Lunar, Madison WI). The potential for bias was controlled by our instructions to the participants that the study involved measuring their energy expenditure during exercise after ingesting and metabolizing two different beverage energy substrates in the form of beetroot and orange juices.

### 2.2. Nitrate Supplementation

Subjects were randomly assigned to consume 500 mL of either beetroot juice (Biotta Inc., Carmel, IN) or the placebo control beverage, orange juice. Our rationale for the 500 mL dose of beetroot juices was based on the acute blood pressure lowering and antiplatelet activity reported at this dose [15]. We selected orange juice as the control because 500 mL had similar constituents as beetroot juice: 220 Cal, 52 g carbohydrates, negligible fat (1 g), 4 g protein, and 140 mg sodium. Orange juice is also similar to beetroot juice in texture and antioxidant content [16] and is reported to have about 5% of the sodium content of beetroot juice [17]. According to the manufacturers' labeling, the sodium content of the orange (Tropicana) and beetroot juices (Biotta) was 0.041 mg/mL and 0.290 mg/mL, respectively, suggesting that the orange juice we used had about 13% of the sodium content of beetroot juice. Biotta beetroot juice has an average listed content of nitrate of 1500 mg/L [18] whereas the nitrate content of orange juice is negligible [19]. Subjects arrived at the laboratory on two separate days, approximately 1-2 weeks apart in a fasting condition. Subjects were instructed to refrain from exercise for 12 h and upon arrival to the laboratory were placed in a sitting position while ingesting the 500 mL beetroot juice or the placebo control orange juice. In order to digest and metabolize the beverages, they were ingested 120 min before the test [18, 20–22]. During the postprandial period, before testing, the subjects remained under supervised resting conditions in the laboratory and did not ingest food or fluid. There were no tolerance issues or untoward effects reported for either the beetroot juice or the control orange juice supplementations. Ingesting beetroot juice did have the benign side effect of producing red urine and stools, which is consistent with previous studies [15, 18, 23]. The strong, albeit benign, effect of beetroot juice on urine color necessitated conducting this study without blinding.

### 2.3. Plasma Nitric Oxide Measurement

Blood samples were collected for measuring the plasma NO concentration 2 h after the beetroot juice and orange juice treatments. Using a sterile lancet, capillary blood (100 *μ*L) was collected from the fingertip. The blood samples were immediately mixed with a nitrite preservation solution containing 0.8 M ferricyanide, 10 mM-N-ethylmaleimide, and 1% NP-40 (Boston BioProducts, Ashland, MA). Following addition of blood to the preservation solution, the sample was deproteinated with trichloroacetic acid (Sigma-Aldrich, St. Louis, MO) and centrifuged at 15000 g for 3 min. The resulting blood plasma solution was then placed into a tube of 1 M sulfuric acid with potassium iodine (Sigma-Aldrich, St. Louis, MO). Blood [NO] was measured in duplicate by an electrochemical method using the inNO-T nitric oxide analyzer (Innovative Instruments, Inc., Tampa, FL). Before each recording, the inNO-T amiNO-700 electrode was calibrated using several dilutions of a standard nitrite solution (100 *μ*M). The performance of the amperometric NO-selective sensor (amino-700XL, Innovative Instruments, Tampa, FL, USA) has been described [24, 25].

### 2.4. Ergometry Tests

All subjects performed a progressive exercise test of VO_2peak_ on an electronically braked leg cycle ergometer (Lode Corival, Groningen, Netherlands). The initial workload began at a level of 20 W for 3 min and was increased by equal work intensities every 3 min to volitional fatigue. Before the study, the cycle ergometer was calibrated for power outputs of 10–1000 W. During the test, blood pressure was determined noninvasively using the SunTech 4240 (SunTech Medical, Inc., Morrisville, NC) automated sphygmomanometric device which measures blood pressure by gating the R-wave with the Korotkoff sounds. Heart rate was measured by an electrocardiogram with three electrodes placed at the RL, LA, and V_5_ anatomical sites connected to the automated blood pressure monitor. In the exercise tests, oxygen consumption (VO_2_), minute ventilation (V_E_), the carbon dioxide excretion rate (VCO_2_), and the respiratory exchange quotient (RQ) were measured breath-by-breath by a computerized metabolic system (Physio-Dyne Max II, Quogue, NY). The VO_2_ measured during the last minute of the progressive exercise test was defined as VO_peak_. Prior to each test, the gas analyzers and respiratory flowmeters were calibrated with high-precision calibration gases (20.99% ± 0.01% O_2_ and 5.00% ± 0.01% CO_2_; Scott Medical Products, Plumsteadville, PA) and a 3-L calibration syringe (Hans Rudolph, Shawnee, KS), respectively. After the VO_2_ test, the subjects performed two separate submaximal ergometry tests under identical conditions on different days, after ingestion of the experimental supplement or control. Five minutes after sitting rest, baseline measures of heart rate, systolic and diastolic blood pressure, and VO_2_ were recorded. The subjects then completed 3 bouts of exercise at the constant submaximal workloads corresponding to 40%, 60%, and 80% of their predetermined VO_2peak_ values with every workload lasting 5 min. The cardiovascular and cerebrovascular responses to the three submaximal aerobic exercise workloads were studied in a stepwise fashion, with no rest intervals in between, by maintaining constant ergometer settings corresponding to 40%, 60%, and 80% of each subject's predetermined VO_2peak_ for 5 min. Heart rate, blood pressure, and VO_2_ were measured at minutes 4 and 5 of each exercise workload, with the mean values used for analysis. The rate-pressure product (heart rate × systolic blood pressure) and mean arterial blood pressure (MABP) were computed. All the submaximal ergometry tests were performed during the luteal phase of the menstrual cycle to eliminate confounding influences of hormonal changes on blood pressure. The heart rate-systolic blood pressure product, a measure of myocardial oxygen demand, was computed.

### 2.5. Cardiac Output Measurement and Computation of Total Vascular (Peripheral) Resistance

Cardiac output was measured noninvasively by using the CO_2_ rebreathing technique. End-tidal partial pressure of CO_2_ (PCO_2_) was measured using a rapid-response infrared CO_2_ analyzer (PHYSIO-DYNE Instrument Corp., Quogue, NY). End-tidal PCO_2_ was used to estimate arterial PCO_2_ [26, 27]. Each subject rebreathed into a 5-1 latex bag containing CO_2_ and O_2_ to permit rapid equilibration with venous PCO_2_. A valid equilibrium during rebreathing was measured by determining a “plateau” in PCO_2_. This criterion requires that, during the first 6–8 s, an inspiration has to be followed by an expiration whose PCO_2_ value is within ±1 mm Hg of the first recorded value. An automated gas mixing apparatus was used to adjust the initial gas volume and initial PCO_2_ in the rebreathing bag. If a trial had to be redone, end-tidal PCO_2_ values were allowed to return to baseline values before the procedure was repeated. Cardiac output was calculated from the indirect Fick equation: CO = *V*_CO_2__/(*C*_*V*CO_2__ − *C*_*a*CO_2__), where CO is cardiac output, *V*_CO_2__ is expired carbon dioxide, *C*_*V*CO_2__ is mixed venous CO_2_ content, and *C*_*a*CO_2__ is arterial CO_2_ content. Total vascular resistance (TVR) was calculated from the following formula: TVR = (MABP)/CO × 80, expressed as dyne·s·cm^−5^ [28].

### 2.6. Transcranial Doppler Ultrasonography: Cerebral Blood Flow Velocity Measurement

Right middle cerebral artery mean blood velocity (MCAV) was measured with transcranial Doppler ultrasonography (Pioneer Series 4040 Transcranial Doppler Ultrasound; Nicolet Vascular, Madison, WI, USA). Ultrasound conductive gel was applied to a 2-MHz transducer probe which was secured to the head (Mark IV, Nicolet Vascular, Madison, WI, USA). Fixation of the ultrasound probe to the measurement area was ensured using a Marc 500 Spencer Probe Fixation System (Spence Technologies, Seattle, WA, USA), which consists of a specialized headgear that has a built-in 2-MHz transducer probe that can be held in a fixed position so that no changes in insonation angle can occur during exercise. The proximal segment of the MCA was insonated through the temporal window approximately 1 cm above the zygomatic arch at a depth of 45–60 mm depending on the cranial thickness. MCAV was defined as the mean of maximum and minimum flow velocities averaged over time throughout the cardiac cycle. Beat-by-beat transcranial Doppler signals were analyzed offline using the 4040 system diagnostic software. The transcranial Doppler ultrasound pulsatility and resistance indexes were recorded. Middle cerebral arterial resistance index (CVRI) was computed as MCAV/mean arterial blood pressure.

### 2.7. End-Tidal Carbon Dioxide Tension (PCO_2_) Measurement

Respiratory gas analysis of end-tidal PCO_2_ on a breath-by-breath basis was measured using an Invivo MAGNITUDE 3150 monitoring system (Invivo Research, Inc., Gainesville, FL, USA). The monitor consists of an infrared CO_2_ analyzer and was calibrated before each study.

### 2.8. Statistical Analysis

Results were expressed as mean ± standard error. A two-way repeated-measures analysis of variance was performed to evaluate the difference between the supplement and control conditions; factor 1 was specified as control orange juice versus beetroot juice treatment and factor 2 as exercise level using SPSS software (SPSS Inc., Chicago, IL). Pearson's product-moment correlation analyses were performed across the plasma [NO] and other study outcome measures. The significance level was set at *P* < 0.05.

## 3. Results

**Figure 1:**
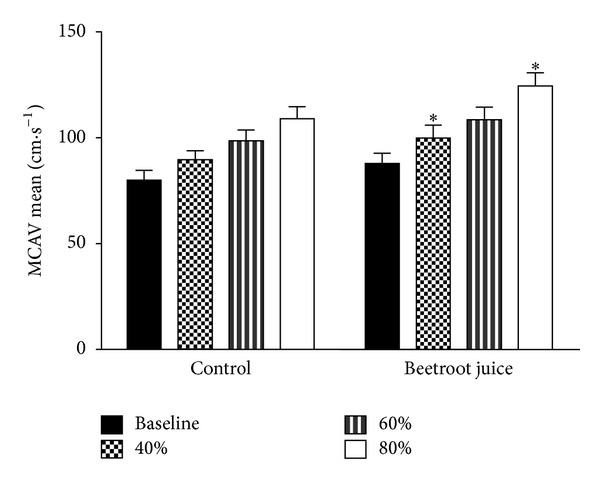
Effects of beetroot juice treatment on middle cerebral arterial blood flow velocity. Bars represent means ± standard errors for middle cerebral artery mean blood flow velocity, expressed in cm·s^−1^. The study subjects were 12 healthy, normotensive young adult African-American females. Left panel: placebo control orange juice treatment. Right panel: beetroot juice treatment. *Difference between control and beetroot juice treatments statistically significant at *P* < 0.05.

Table 1 presents the demographic and anthropomorphic characteristics of the study population.  Figure 1 shows the progressive physiological increases in MCAV from baseline (rest) to the constant exercise workloads set to 40%, 60%, and 80% of the predetermined VO_2peak_, associated with the placebo control orange juice and beetroot juice treatments. These MCAV increments are indicative of normal autoregulation of middle cerebral arterial smooth muscle. The beetroot juice treatment is shown to be associated with significantly greater MCAV than that of the orange juice treatment only at the workloads set to 40% and 80% of the predetermined VO_2peak_. The end-tidal carbon dioxide tensions (PCO_2_) were not significantly different between the control and beetroot juice treatment conditions at rest as well as at the three levels of aerobic exercise studied (not shown).

**Figure 2:**
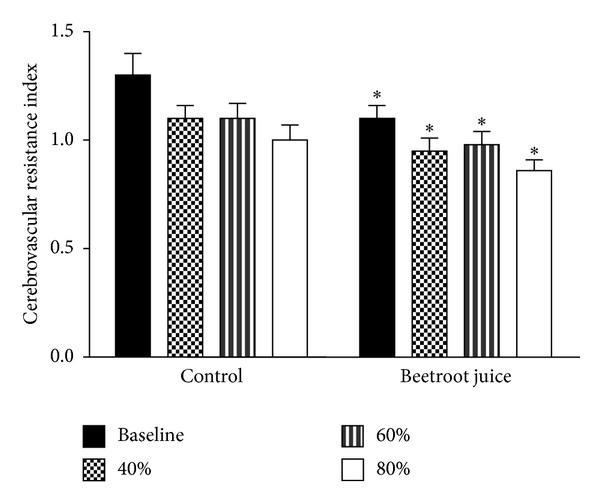
Effects of beetroot juice treatment on middle cerebral arterial cerebrovascular resistance index. Bars represent means ± standard errors for middle cerebrovascular resistance index, computed as the ratio mean arterial blood pressure/mean middle cerebral artery blood flow velocity, expressed in mm Hg·cm^−1^·s^−1^. The study subjects were 12 healthy, normotensive young adult African-American females. Left panel: placebo control orange juice treatment. Right panel: beetroot juice treatment. *Difference between control and beetroot juice treatments statistically significant at *P* < 0.05.

Figure 2 shows the physiological increases in CVRI from rest and during aerobic exercise after the control orange juice and beetroot juice treatments, reflecting normal regulation. After the beetroot juice treatment, significantly lower CVRI was observed at rest and also at the three levels of aerobic exercise studied.

Table 2 shows the effects of the control and experimental beetroot juice treatment on the baseline plasma [NO] and on the baseline cardiovascular variables measured at rest. Plasma [NO] was significantly greater after the beetroot juice than after the placebo orange juice control treatment. Correlations between plasma [NO] and all the other measured variables were not significant. The systolic and mean arterial blood pressure and the heart rate-systolic pressure product (RPP) were found to be significantly lower after the beetroot juice treatment.

Table 3 presents the effects of the beetroot juice treatment on the cardiovascular parameters measured during aerobic exercise on a cycle ergometer at constant workloads corresponding to 40%, 60%, and 80% of each subject's predetermined VO_2peak_. Systolic arterial pressure and the heart rate-systolic pressure product were lower after the beetroot juice treatment at the 40%, 60%, and 80% VO_2peak_ workloads; mean arterial blood pressure was lower only at the 40% VO_2peak_ workloads. The beetroot juice treatment decreased the whole body VO_2_ at the three levels of exercise studied (*P* < 0.05). There were no treatment-related differences in the VO_2_ measured at rest.

**Figure 3:**
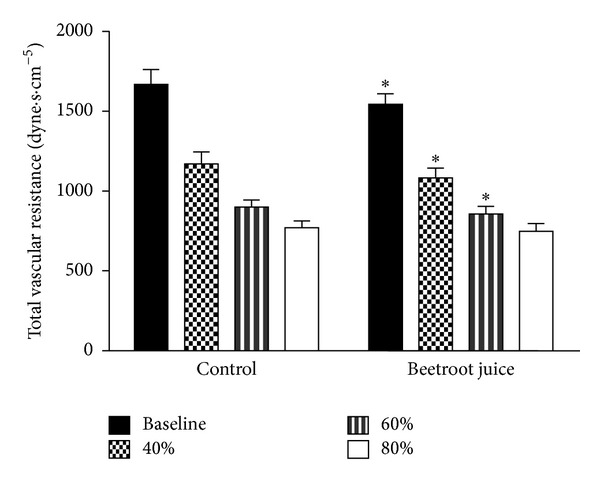
Effects of beetroot juice treatment on total vascular resistance. Bars represent means ± standard errors for total vascular (peripheral) resistance computed as the ratio mean arterial blood pressure/cardiac output, expressed in dyne·s·cm^−5^. The subjects were 12 healthy, normotensive young adult African-American females. Left panel: placebo control orange juice treatment. Right panel: beetroot juice treatment. *Difference between control and beetroot juice treatments statistically significant at *P* < 0.05.

Figure 3 shows the progressive physiological increments in total vascular resistance (TVR) from rest to the three constant exercise workloads studied after the control orange juice treatment. After the beetroot juice treatment, significantly lower TVR was observed at rest, as well as at the 40% and 60% VO_2peak_ exercise workloads but not at the 80% VO_2peak_ workload.

Table 4 shows the effects of the control and beetroot juice treatments on the transcranial Doppler ultrasound pulsatility and resistance indexes measured at rest and at the three constant exercise workloads studied. The four activity levels produced progressive, physiological changes (increases) in the pulsatility and resistance indexes but the treatment-related differences in these indexes were found to be not significant.

Table 5 summarizes the treatment-related correlations between MCAV and each of the important cardiovascular variables measured and computed, across the four conditions studied (baseline rest and three submaximal levels of aerobic exercise). The correlations between MCAV and mean arterial blood pressure were not significant. The control, beetroot juice treatment-related correlations between MCAV and cardiac output (*r* = +0.45,  +0.46), between MCAV and TVR (*r* = +0.52,  +0.43), and between MCAV and CVRI (*r* = −0.89,  −0.89) were found to be significant at *P* < 0.01 to *P* < 0.001.

## 4. Discussion

The main finding of this study is that, compared to a placebo orange juice control treatment, a single beetroot juice treatment decreased CVRI at rest and at three submaximal exercise workloads. Because the sample size of this study and the effects were small, the results of this study are preliminary and definitive conclusions must, therefore, await confirmation by an expanded study. However, if these small, significant treatment-related differences in CVRI in healthy subjects were to occur over a prolonged period of time, the effects could be amplified by a factor of dose × time. Moreover, if these effects were to occur in patients with compromised neurological or cardiovascular functions, they may be able to counteract diseases complicated by increased CVRI. The treatment-related effects described herein occurred in the absence of treatment-related differences in end-tidal PCO_2_ which are known to affect TCD measurements significantly [29].

Although significant beetroot juice treatment-related changes (increases) in MCAV were restricted to 40% and 80% of the predetermined VO_2peak_ workloads, beetroot juice treatment-related decrements in computed CVRI were detected at rest and at the 40%, 60%, and 80% VO_2peak_ workloads. Thus, the overall impact of beetroot juice on MCAV remains unclear. Nevertheless, at rest and at the 60% workload, CVRI and TVR were found to be significantly lower after the beetroot juice than after the control treatment. This lower CVRI and TVR occurred in the absence of a treatment-related difference in MCAV. This pattern, wherein MABP was lower in the absence of a change in MCAV, is similar to that described for patients with postprandial [30] and neurogenic [31] syncope. This response pattern may be explained by relaxation of muscular conduit arteries rather than arteriolar resistance vessels, known effects of nitric oxide donor molecules and the vasodilator drug nitroprusside [32]. The negative finding with respect to MCAV suggests that the treatment-related (lower) CVRI after beetroot juice likely resulted from nitrate-induced lowering of MABP and TVR. These responses were likely below the threshold for a sympathetic neural response that would have constricted middle cerebral arteries and/or arterioles and would have increased MCAV. Such sympathetic neural regulation was evidenced by increased cerebral vascular resistance to maintain constant cerebral perfusion as was demonstrated by the physiological, exercise-related increases in MCAV. In other studies, increases in MCAV associated with sympathetic vasoconstriction are shown to occur during both exercise and cold pressor testing [33–35]. Although it was beyond the scope of this study to measure the amounts of sympathetic cardiovascular modulation, future studies could employ heart rate variability measurements of the sympathetic influences on heart rate to determine whether the beetroot juice treatment could be an effective modulator of autonomic signaling activity.

At the 40% workload, CVRI and TVR were also found to be significantly lower after the beetroot juice treatment, but with greater MCAV than those after the control orange juice treatment. This finding suggests that nitrate-induced vasorelaxation was likely associated with the aforementioned sympathetic neural response that seemed to be absent at rest and at the 60% workload. One explanation for greater MCAV after the beetroot juice treatment is middle cerebral arterial vasoconstriction, which has been described for exercise-induced increases in blood pressure [33]. Another explanation for greater MCAV is greater blood flow secondary to greater cardiac output. A mechanism for nitrate-induced increases in cardiac output has not been described. RPP is an indicator of myocardial oxygen demand and lower RPP occurred after the beetroot juice in this and in a previous study [13]. Such nitrate-induced lowering of RPP indicates that oxygen utilization was less at the same workloads and there were significant positive correlations between MCAV and cardiac output across the experimental conditions for both treatments. The mechanisms for greater MCAV after the beetroot juice treatment may, therefore, be a specific effect of sympathetic neural stimulation of cerebral arteries and should be clarified in future studies.

At the 80% workload, MCAV was also found to be higher and CVRI lower after the beetroot juice treatment, but without a treatment-related difference in TVR. Unlike the results at the 40% workload, higher MCAV and lower CVRI occurred in the absence of generalized systemic vasorelaxation, which may have been exhausted at this (relatively high) workload.

All the treatment-related decrements in CVRI occurred in the absence of treatment-related changes in the TCD-measured pulsatility and resistance indexes. This finding implies a difference between these TCD indexes and physiologically computed CVRI. Physiologically computed CVRI takes into account the dynamic aspects of blood pressure and the TCD-measured pulsatility and resistance indexes do not. These findings suggest that, when the experimental changes or differences are small, the TCD-measured pulsatility and resistance indexes may be less sensitive than physiologically computed CVRI to the treatment-related effects described herein.

The beetroot juice treatment-related decrements in the whole body VO_2_ that we observed only during aerobic exercise suggest that this dietary nitrate might have had an effect on intracellular VO_2_ regulating mechanisms in skeletal muscle mitochondria. Indeed, improved efficiency of skeletal muscle oxygenation has been reported with nitrate supplementation [36]. Moreover, oxidation of NO and nitrate to nitrite is reported to oxidize cytochrome c, thereby providing a mechanism for more efficient oxygen utilization by enhancing oxidative phosphorylation [37]. The lack of correlation of the blood NO levels with the cardiorespiratory and hemodynamic measurements in this study is consistent with reports that plasma nitrate and nitrite concentrations may also play important roles in the blood pressure lowering activity of dietary supplemented nitrates [20]. Thus, as we have shown, although NO appears to be a reliable physiological marker for dietary nitrate supplementation, the plasma nitrate and nitrite concentrations are also shown to affect the activity of endothelial NOS and, therefore, the bioavailability of NO [20].

Our results are consistent with those of Presley et al. who performed a study of elderly, more-than-70-year-old subjects on high (12 mM) and low (0.09 mM) nitrate diets for several days that included but was not restricted to beetroot juice as sources of nitrates [38]. The high-nitrate diet increased blood flow, measured by arterial spin labeling magnetic resonance imaging, to frontal cortex white matter between the dorsolateral prefrontal and anterior cingulate cortexes, in the absence of a global change in cerebral blood flow [38]. Although the present study did not directly address the efficacy of beetroot juice as an adjunctive dietary treatment for brain hypoperfusion syndromes, these findings appear to imply the potential for efficacy as such. Compared to nondemented age-matched controls, Alzheimer's disease patients exhibit higher CVRI associated with lower MCAV and higher MABP, and TVR [39]. In the present study, lower CVRI was associated with unchanged or higher MCAV, lower MABP and lower TVR at rest and at three submaximal exercise workloads after a single beetroot juice treatment. These findings are opposite to those of the aforementioned Alzheimer's study [39] and, therefore, suggest that dietary nitrate supplementation might have the potential to counteract these pathophysiological features of Alzheimer's disease.

### 4.1. Critique of Methods and Limitations of Study

It is common for NO to be measured as NO metabolites, which include the concentrations of its stable oxidation products, nitrate and nitrite ions. Because NO bioavailability is thought to be the key issue in endothelial function, we measured NO directly by an electrochemical method. Takarada et al. reported measurements of NO by the same method and validated it using another methodology, the Griess reaction, which detects nitrite based on the reaction with sulphanic acid to form the diazonium ion [25]. A significant correlation was found between the NO levels measured by the electrochemical NO sensor and the nitrite level measured by the Griess reaction (*r* = 0.41, *P* = 0.048) [25]. Takarada et al. also used saline as a control marker of the NO sensor and reported that infusion of saline did not cause any change in the plasma NO concentration [25]. In addition, no significant changes in the baseline current were observed with and without mixing, suggesting no direct effect of fluid (blood) motion on the NO sensor measurement. Nitrate-depleted beetroot juice might have been a better control than orange juice. Also, the laboratory personnel could have been blinded to the allocation. However, our experimental endpoints involved computing systemic and cerebral vascular resistances at rest and during aerobic exercise at light, moderate, and heavy fixed workloads. Because of the autonomic, involuntary nature and multiple sources of reflex inputs to the variables of interest, it was considered doubtful that the subjects and/or the laboratory personnel would have been able to systematically bias the results of blood pressure/cardiac output or middle cerebral arterial blood flow measurements under these conditions. It was beyond the scope of our study to measure the precise concentration of nitrate in the beetroot juice. Ours was a physiological study to determine whether beetroot juice possessed vasodilator activity sufficient to alter both systemic and cerebral hemodynamics in healthy young adult African-American females. We used blood NO, an endogenous vasodilator molecule, as a biochemical marker for beetroot juice activity in that regard. Nevertheless, a limitation of the present study is that we did not measure the concentration of nitrates in the beetroot juice; rather, we relied on the manufacturer's specifications. Thus, there could be variability in the nitrate concentration of different beetroot juice preparations that obviates our ability to compare our results to those of other studies. For example, if the manufacturer's labeling is accurate, 1500 mg/L provided about 12 millimoles of nitrate in 500 mL of juice in our study. If the labeling is accurate, this concentration of nitrate is the same as that provided to a study group of healthy elderly (more-than-70-year-old) subjects studying cerebral perfusion by methods different from ours [38]. In that study, regional cerebral blood flow was increased following several days of a high-nitrate (12 mM) diet that included beetroot juice as the main high-nitrate component, compared to a control low-nitrate diet consisting of 0.09 mM nitrate. For comparison, the concentration of nitrate used in a study by Webb et al. [15] was 45 mM providing 22.5 millimoles in the 500 mL of juice given, almost twice that of ours. The concentration of nitrate in our study was likely to have been even less than the 18 mM beetroot juice used by Kenjale et al. [18]. Considering the potential variability, we cannot make any conclusions about the relative potency of vasodilator activity in the beetroot juice. This difficulty is underscored by the facts that the subjects in the Kenjale et al. study were elderly males and females of both Caucasian and African ethnicities diagnosed with peripheral arterial disease wherein the main blood pressure finding was a beetroot juice treatment-related decrement in resting and exercising diastolic blood pressure [18]. For comparison, the Webb study subjects were healthy male and female volunteers with treatment-related decrements in resting systolic and diastolic blood pressure in the same timeframe as ours. Our healthy young adult subjects exhibited resting systolic blood pressure decrements in the absence of treatment-related diastolic blood pressure differences. Gender differences in the endogenous handling of nitrate have been demonstrated. The same dietary nitrate load is reported to have produced small differences in plasma nitrate levels between males and females but the plasma nitrite concentrations of the female subjects were found to be two-fold greater than those of the male subjects [20]. The subjects in the present study were all females, perhaps providing an explanation for the small, yet significant, blood pressure changes. These findings suggest that a similar study in males might produce larger physiological changes in blood pressure, systemic vascular, and cerebral vascular resistances. We also did not measure other physiologically active constituents of the treatments such as sodium. Thus, constituents other than nitrates or nitrites (e.g., sodium) could have played a role in the physiological responses reported in this study. According to the manufacturers' labeling, the sodium content of the orange juice used should have been about 13% of that in the beetroot juice. The substantially greater level of sodium in the beetroot juice would be expected to raise blood pressure, perhaps explaining why we did not observe larger beetroot juice treatment-related decrements in blood pressure.

## 5. Conclusions

In summary, the results of this study demonstrate that a dietary nitrate treatment with beetroot juice, which increased the plasma nitric oxide concentration quite significantly, decreased a Doppler ultrasonographic measure of middle cerebral arterial blood flow velocity and a physiologically computed index of cerebrovascular resistance. This beetroot juice treatment-related change in cerebrovascular resistance index was associated with decrements in systolic blood pressure and the rate-pressure product index of myocardial oxygen demand. Because the plasma nitric oxide concentration was not correlated with any of these variables, the plasma nitric oxide level may be a biochemical marker rather than a direct causative agent for these findings. The findings of this study suggest that the physiological mechanisms underlying dietary nitrate-induced improvements in systemic and cerebral vascular hemodynamics may be somewhat workload dependent. The hemodynamic improvements associated with the beetroot juice treatment appear to be consistent with counteracting some of the pathophysiologic cerebral vascular features of hypertension, Alzheimer's, and other diseases associated with brain hypoperfusion and cognitive deficits.

## Figures and Tables

**Table 1:** Descriptive characteristics of the study subjects.

Characteristics	Subjects (*n* = 12)
Age (yr)	20.7 ± 0.3
Height (cm)	160.9 ± 1.6
Weight (kg)	61 ± 2.4
Body fat (%)	27.9 ± 3.7
VO_2peak_ (mL·kg^−1^·min^−1^)	26.1 ± 1.3

Data are means ± standard errors. VO_2peak_ = peak oxygen consumption.

**Table 2:** Effects of beetroot juice treatment at rest.

Variable	Control	Beetroot juice
Nitric oxide (nM)	4.5 ± 1.0	17.8 ± 2.6*
Cardiac output (L·min^−1^)	4.8 ± 0.1	4.9 ± 0.1
Oxygen consumption (mL·kg^−1^·min^−1^)	4.5 ± 1.0	4.0 ± 0.5
Systolic blood pressure (mm Hg)	119.8 ± 2.8	114.8 ± 2.1*
Diastolic blood pressure (mm Hg)	90.0 ± 2.4	86.4 ± 1.3
Mean blood pressure (mm Hg)	99.6 ± 2.4	95.3 ± 1.4*
Heart rate (b·min^−1^)	85.8 ± 3.2	82.5 ± 3.0
Heart rate-systolic pressure product	10,785 ± 414	9,751 ± 226*

Values expressed in mean ± standard error, measured at rest (baseline). *Significantly different from control at *P* < 0.05.

**Table 3:** Effects of beetroot juice treatment at submaximal exercise.

Variable	Control	Beetroot juice
	40% VO_2peak_	
Cardiac output (L·min^−1^)	6.6 ± 0.3	6.8 ± 0.2
Oxygen consumption (mL·kg^−1^·min^−1^)	12.2 ± 2.0	9.5 ± 2.5*
Systolic blood pressure (mm Hg)	129 ± 3.3	120.9 ± 4.1*
Diastolic blood pressure (mm Hg)	82 ± 1.9	79.9 ± 2.1
Mean blood pressure (mm Hg)	97.3 ± 1.9	93.0 ± 2.5*
Heart rate (b·min^−1^)	104.8 ± 3.0	102.0 ± 2.9
Heart rate-systolic pressure product	13,452 ± 573	12,367 ± 641*

	60% VO_2peak_	
Cardiac output (L·min^−1^)	9.3 ± 0.3	9.6 ± 0.4
Oxygen consumption (mL·kg^−1^·min^−1^)	17.5 ± 2.1	15.0 ± 2.4*
Systolic blood pressure (mm Hg)	148.4 ± 4.0	141.9 ± 3.3*
Diastolic blood pressure (mm Hg)	85.4 ± 1.5	85.0 ± 1.9
Mean blood pressure (mm Hg)	105.9 ± 2.4	103.6 ± 2.1
Heart rate (b·min^−1^)	132.6 ± 2.9	130.4 ± 3.2
Heart rate-systolic pressure product	19,785 ± 912	19,264.1 ± 819*

	80% VO_2peak_	
Cardiac output (L·min^−1^)	11.3 ± 0.6	11.4 ± 0.6
Oxygen consumption (mL·kg^−1^·min^−1^)	22.6 ± 3.0	19.5 ± 3.3*
Systolic blood pressure (mm Hg)	162.8 ± 4.6	151.6 ± 4.3*
Diastolic blood pressure (mm Hg)	87.5 ± 3.7	84.4 ± 2.1
Mean blood pressure (mm Hg)	109.9 ± 2.8	107.0 ± 2.1
Heart rate (b·min^−1^)	154.5 ± 2.8	153.0 ± 3.0
Heart rate-systolic pressure product	25,337 ± 889	23,146 ± 754*

Values expressed in mean ± standard error measured at rest and at constant aerobic exercise workloads corresponding to 40%, 60% and 80% of subject's predetermined peak oxygen consumption (VO_2peak_). *Significantly different than control at *P* < 0.05.

**Table 4:** Effects of beetroot juice treatment on TCD pulsatility and resistance.

Variable	Control	Beetroot juice
	Pulsatility index	
Baseline rest	0.75 ± .04	0.71 ± .03
40% VO_2peak_	0.93 ± .05	0.87 ± .04
60% VO_2peak_	0.93 ± .03	0.94 ± .03
80% VO_2peak_	1.06 ± .07	1.08 ± .05

	Resistance index	
Baseline rest	0.49 ± .01	0.47 ± .01
40% VO_2peak_	0.56 ± .02	0.54 ± .01
60% VO_2peak_	0.57 ± .01	0.56 ± .01
80% VO_2peak_	0.60 ± .02	0.60 ± .01

Values expressed in mean ± standard error of pulsatility and resistance indexes measured by transcranial doppler (TCD) ultrasonography at rest and at constant aerobic exercise workloads corresponding to 40%, 60%, and 80% of subject's predetermined peak oxygen consumption (VO_2peak_).

**Table 5:** Treatment-related correlations across conditions.

Correlations	Control	Beetroot juice
MCAV versus MABP	+0.13 (NS)	+0.21 (NS)
MCAV versus CO	+0.45*	+0.46*
MCAV versus TVR	−0.52**	+0.43*
MCAV versus CVRI	−0.89**	−0.89**

MCAV: middle cerebral arterial blood flow velocity; MABP: mean arterial blood pressure; CO: cardiac output; TVR: total vascular (peripheral) resistance; CVRI: cerebral vascular resistance index.

Values are Pearson's product-moment correlation coefficients computed across baseline rest and three submaximal aerobic exercise conditions.

NS: not statistically significant.

*Significant at *P* < 0.01, **significant at *P* < 0.001, 46 df.
